# Changes in amino acids, catechins and alkaloids during the storage of oolong tea and their relationship with antibacterial effect

**DOI:** 10.1038/s41598-024-60951-5

**Published:** 2024-05-07

**Authors:** Jilai Cui, Bin Wu, Jie Zhou

**Affiliations:** 1 College of Life Science, Xinyang Normal University, 237 Nanhu R., Xinyang, 464000 Henan People’s Republic of China; 2 State Key Laboratory of Tea Plant Biology and Utilization, Anhui Agricultural University, 130 Changjiang Ave W., Hefei, 230036 Anhui People’s Republic of China

**Keywords:** Nutrition, Antifungal agents

## Abstract

The storage process has a significant impact on tea quality. Few is known about effect of storage on quality of oolong tea. This study aimed to assess the effect of different storage times on the key chemical components of oolong tea by measuring changes in catechin, free amino acid, and alkaloid content. Variation in the main substances was determined by principal component analysis and heat map analysis. The results revealed notable effects of the storage process on the levels of theanine, epigallocatechin gallate (EGCG), and glutamine. These findings suggest that these compounds could serve as indicators for monitoring changes in oolong tea quality during storage. Additionally, the study observed an increase in the antibacterial ability of tea over time. Correlation analysis indicated that the antibacterial ability against *Micrococcus tetragenus* and *Escherichia coli* was influenced by metabolites such as aspartic acid, threonine, serine, gamma-aminobutyric acid, ornithine, alanine, arginine, and EGCG. Overall, this study presents an approach for identifying key metabolites to monitor tea quality effectively with relatively limited data.

## Introduction

The increasing popularity of tea can be attributed to its unique flavor and health benefits, which stem from its rich content of natural compounds. In general, free amino acids play a key role in imparting the umami taste to tea, with taste intensity increasing alongside the concentration of amino acids^1,2^. Theanine, which typically constitutes approximately 0.2–2% of the dry weight, is the most prevalent amino acid in tea, contributing to around 60–70% of the total free amino acid content in tea leaves^3–5^. Tea is rich in catechins and alkaloids, which contribute to astringency and bitterness, respectively^6^. Due to the high content of these compounds, tea has many health benefits including antioxidant^7,8^, antimicrobial^9,10^, anti-cancer^11^, and anti-inflammatory activities^12,13^.

Previous research has demonstrated that the quality of green tea, including aroma, overall tea quality, and biological value, notably deteriorates with prolonged storage time^14^. Additionally, increased storage durations lead to a reduction in the polyphenol content, the primary bioactive compounds in white tea, potentially resulting in decreased bioactivities associated with them^15^. However, storage is an essential condition for the formation of the quality of pu-erh tea. Pu-erh tea with longer storage times is usually more highly prized in the tea market^16^. Some studies have revealed that the concentrations of amino acids and flavonoids decreased while those of gallic acid and lipids increased in a time-dependent manner for some of the main metabolites of raw pu-erh tea^17,18^. Oolong tea is commonly stored for weeks or months before consumption. Few is known about the dynamic changes in the principal chemical and aroma components relevant to the quality of oolong tea during storage.

Due to the significant stress of contemporary lifestyles, numerous individuals encounter inflammation. Several compounds in tea possess anti-inflammatory properties^19^. Inflammation constitutes the body's innate immune response to detrimental stimuli, such as tissue damage and pathogenic intrusion, serving as a vital defense mechanism against threats. Despite their distinct etiologies, conditions like arthritis, asthma, and chronic obstructive pulmonary disease all stem from dysregulated inflammatory responses. Prolonged inflammation can precipitate various diseases, encompassing cancer and rheumatoid arthritis^20^. *Micrococcus tetragenus*, a strain of Gram-positive bacteria, has been linked to inflammation-associated ailments like pneumonia, endocarditis, sepsis, and meningitis^21,22^. Pathogenic *Escherichia coli*, a Gram-negative bacterium^23^, is also implicated in eliciting inflammation, particularly in cases of gastroenteritis^24,25^. Both pathogens can instigate inflammation in the human body.

Tea polyphenols, which are main health-promoting compounds in tea, have garnered significant interest. Numerous recent studies have demonstrated the strong antioxidant activity of catechins, and other polyphenols found in tea^26,27^. These compounds exhibit antioxidant effects in vitro by scavenging nitrogen and reactive oxygen species, generated from various oxidative stresses, and chelating metal ions^28,29^. Additionally, tea polyphenols may reduce the risk of cardiovascular disease and certain cancers, improve physiological functions, such as reducing blood pressure, increasing bone density, and managing weight^30,31^. Furthermore, they exhibit considerable potential as antibacterial agents, being effective against both Gram-positive and Gram-negative pathogens^32,33^. Among these, epigallocatechin gallate (EGCG), the primary component of green tea, has been found to possess the highest biological activity^34^, including anti-inflammatory effects. Green tea and EGCG inhibit the expression of inflammatory cytokines, inflammation-related enzyme genes, and proteins^20^. The robust antioxidant activity and health benefits of EGCG and green tea have received noteworthy attention in recent years^35^.

Other tea extract exhibits a bactericidal effect on *Staphylococcus aureus* and *Escherichia coli*^36^. This extract contains high concentrations of EGCG, and epigallocatechin (EGC) displays potent antibacterial activity against bacterial pathogens^37^. Additionally, amino acids are crucial for preventing and treating intestinal inflammation, improving intestinal barrier function, and modulating the expression of anti-inflammatory cytokines and tight junction proteins, while also reducing oxidative stress, intestinal cell apoptosis, and pro-inflammatory cytokines in intestinal inflammation^38^. Furthermore, studies have indicated that glutamate signaling played a regulatory role in the bone remodeling system and joint tissues, influencing the processes of inflammation and injury in arthritis^39^.

Oolong tea extract has been found to reduce vascular inflammation in mice fed with carnitine^40^, while its theaflavins^41^ and theophylline^12^ also exhibit anti-inflammatory effects. However, the anti-inflammatory and antibacterial abilities of oolong tea during storage have not been thoroughly studied. It is known that during tea storage, the composition of tea polyphenols, soluble sugars, and other components undergoes changes, which serve as the material basis for the anti-obesity and anti-inflammatory effects of oolong tea. Moreover, the physiological effects of oolong tea are believed to undergo changes during storage^42^. Therefore, this study aimed to investigate the changes in the antioxidant and antibacterial properties of Wuyi rock tea during storage.

## Materials and methods

### Plant materials and treatments

The tea cultivars (*Camellia sinensis* var. *sinensis* “Beidou”) were provided by YanHuang tea plantation in the Wuyi Mountains, Fujian, China (27°31′43″ N, 117°04′48″ E). Beidou oolong tea samples were made under the traditional oolong tea manufacturing process, including withering, making, fixing, rolling, roasting (twice), and full fire processing by an experienced tea master. The process utilized freshly plucked tea leaves donated by ManTingFeng Tea Co., Ltd. in Wuyi Mountain City, China. The tea was produced in May 2018 and subsequently stored at − 80 °C to avoid possible chemical change before treatment. Then the tea was subjected to storage treatment by storing at 25 °C. The tea samples stored at 25 °C for 2, 4, 8, 12 and 16 weeks were collected and named after 2w, 4w, 8w, 12w and 16w, respectively. Tea sample without storage was used as control (ck). Then the samples were freeze-dried, grounded, and passed through a 0.35 mm sieve.

### Chemicals

Distilled water was produced using a Milli-Q water purification system (Millipore, Billerica, MA, USA). Methanol of LC–MS grade was purchased from Merck (Darmstadt, Germany). Ammonium acetate, formic acid, gallic acid (purity ≥ 99%), gallocatechin (GC, purity ≥ 99%), caffeine (CAF, purity ≥ 99%), theobromine (purity ≥ 99%), EGC (purity ≥ 99%), catechin (C, purity ≥ 99%), EGCG (purity ≥ 99%), epicatechin gallate (ECG, purity ≥ 99%), and epicatechin (EC, purity ≥ 99%) were obtained from Sigma-Aldrich (St. Louis, MO). The following free amino acids were obtained from Sigma-Aldrich: aspartic acid (Asp), l-threonine (Thr), l-serine (Ser), l-glutamic acid (Glu), alpha-aminoadipic acid (α-AAA), glycine (Gly), l-alanine (Ala), citrulline (Cit), α-amino-n-butyric acid (α-ABA), l-valine (Val), l-cysteine (Cys), l-isoleucine (Ile), l-leucine (Leu), l-tyrosine (Tyr), l-phenylalanine (Phe), β-alanine(β-Ala), γ-aminobutyric acid (GABA), l-ornithine (Orn), l-histidine (His), l-arginine (Arg), l-proline (Pro), glutamine (Gln) and theanine (Thea). Total antioxidant capacity was detected by a total antioxidant capacity assay kit with the 2,2-azino-bis(3-ethylbenzthiazoline-6-sulfonic acid) (ABTS) method (Solarbio Science, Beijing, China), 1,1-diphenyl-2-picrylhydrazyl (DPPH) method (Solarbio Science, Beijing, China), and ferric reducing antioxidative potential (FRAP) method (Solarbio Science, Beijing, China), respectively.

### Antibacterial ability of WRT

The method for evaluating antibacterial activity was in accordance with a previous study^43^. To prepare tea infusion, 50 g of tea powder were added to 200 mL of boiling water and extracted for 10 min in a 70 °C water bath. Then the solution was filtered to remove solid. Next, 100 μL of tea soup was added to 100 μL of bacterial solution with OD600 about 0.6–0.8 activity in a 96-well plate. The mixture was incubated under 37 °C for 48 h and a microplate reader recorded the absorption at 600 nm every 2 h. The mixture of 100 μL bacterial solution and 100 μL Luria–Bertani (LB) medium was used as positive control and the mixture of 100 μL tea infusion and 100 μL LB medium was used as negative control.

### Antioxidant activity of WRT

To prepare tea infusion, 0.01 g of the ground sample was placed in a centrifuge tube, and 400 μL of distilled water was added. After extracting for 7 min at 90 °C, the extract was filtered an 0.45 μm membrane. The tea infusion was used for further experiments.

For DPPH method, the tea sample extract was diluted 25 times. Then different volumes (5, 10, 15, and 20 μL) of diluted solution were added to the tube and adjusted up to 100 μL with distilled water, respectively. After mixing, the above solutions were mixed with 100 μL of DPPH solution (0.1 mM). The mixture was incubated at room temperature for 30 min in the dark. Distilled water was used as blank. The absorbance was measured at a wavelength of 517 nm^44^.

For FRAP method, 5 μL of tea extract solution was added to 180 μL of FRAP working solution. After incubation at 37 °C for 5 min, the absorbance at a wavelength of 593 nm was measured. Distilled water was used as control. FeSO_4_ solutions at different concentrations were used as standards to generate a calibration curve and the FRAP antioxidant activity was expressed in millimolar FeSO_4_ per liter of tea extract solution (mM FeSO_4_)^45^.

For ABTS assay, the tea sample extract was diluted 25 times. Diluted solutions of different volumes (5, 10, 15, and 20 μL) were added to the tube and adjusted up to 100 μL with distilled water. Each solution was added to 100 μL of ABTS working solution. After incubated for 6 min at room temperature in dark, the absorbance of mixture was measured at a wavelength of 734 nm^44^. Distilled water was used as control and results were expressed as the inhibition percentage of ABTS.

### Analysis of free amino acids

The free amino acid content was analyzed using a high-speed amino acid analyzer (L-8900, Hitachi) according to a previous study^46^. Briefly, 0.1 g of tea sample powder was placed into a flask, extracted with 4 mL of 4% sulfosalicylic acid, ultrasonically extracted for 30 min, and then centrifuged at 13,680*g* for 40 min. The precipitate was extracted again with the same procedure. The supernatant was combined and adjusted to 8 mL with distilled water, and 1 mL of the supernatant was filtrated through a 0.22-μm water membrane before analysis. Lithium citrate solution was used as mobile phase and ultraviolet–visible (UV–Vis) detection wavelengths were 570 and 440 nm. The flow rates were 0.35 mL/min for the mobile phase and 0.3 mL/min for the derivatization reagent. The column temperature was set to 38 °C, and the post-column reaction equipment was maintained at 130 °C. The temperature of the autosampler was kept at 4 °C, and the injection volume was 20 μL. The peak areas of compounds were compared with the amino acid standards. All measured compounds were used in three replicates, and the results were presented as mean ± standard deviation (SD).

### Analysis of catechins, alkaloids, and gallic acid by HPLC

The contents of catechins, alkaloids and gallic acid were analyzed by HPLC according the previous study with small modifications^47^. First, 0.1 g of tea sample powder was placed into the flask, extracted with 1 mL of 70% methanol, and shaken for 30 min, followed by cryogenic ultrasound extraction for 20 min and centrifugation (16,000 rpm/min, 15 min, 4 °C). The precipitate was subjected to the same extraction procedure once more. The supernatant was combined and adjusted to 2 mL with distilled water and filtrated through a 0.22 μm water membrane before analysis. A DIONEX UltiMate 3000 UHPLC system (Thermo Fisher Scientific, MA, USA) with an autosampler was used for liquid chromatography. Separation was performed on a reverse-phase Zorbax Eclipse plus a C18 column (1.8 μm, 100 × 2.1 mm, Agilent Technologies, Little Falls, DE) with a solvent flow rate of 0.2 mL/min at a column temperature of 40 °C. Distilled water with 0.1% (v/v) formic acid was used as solvent A and acetonitrile with 0.1% (v/v) formic acid was used as solvent B. The solvent gradient was as follows: 0–2 min 5% B, 2–3 min linear ramp to 8% B, 3–7 min linear ramp to 12% B, 7–13 min linear ramp to 14% B, 13–15 min linear ramp to 40% B, 15–17 min linear ramp to 95% B, 17–20 min 90% B, 20–20.5 min linear ramp to 5% B, and 20.5–23 min 5% B. The injection volume was 1 μL and, UV detection was performed at 275 nm. Catechins, alkaloids, and gallic acid in samples were identified by comparing the retention time with that of standards, and quantitated with the corresponding calibration curves equation of their corresponding calibration curves. The results were expressed as mean ± SD based on the dry weight with n = 3.

### Statistical analysis

Excel (Microsoft Office Standard 2019, Microsoft Corporation, Redmond, WA, USA) was used to calculate the concentration of each component. The heatmaps were performed in TBtools-II v2^48^. Principal component analysis (PCA) plots were performed using SIMCA 14.1 (Umetrics, Umeå, Sweden). The results were expressed as mean ± standard deviation (SD) based on the dry weight (DW) of the samples with n = 3.

### Ethics approval and consent to participate

We confirm that all the experimental research and field studies on plants (either cultivated or wild), including the collection of plant material, complied with relevant institutional, national, and international guidelines and legislation.

## Results and discussion

### Antibacterial effect and antioxidant capacity of tea during storage

**Figure 1:**
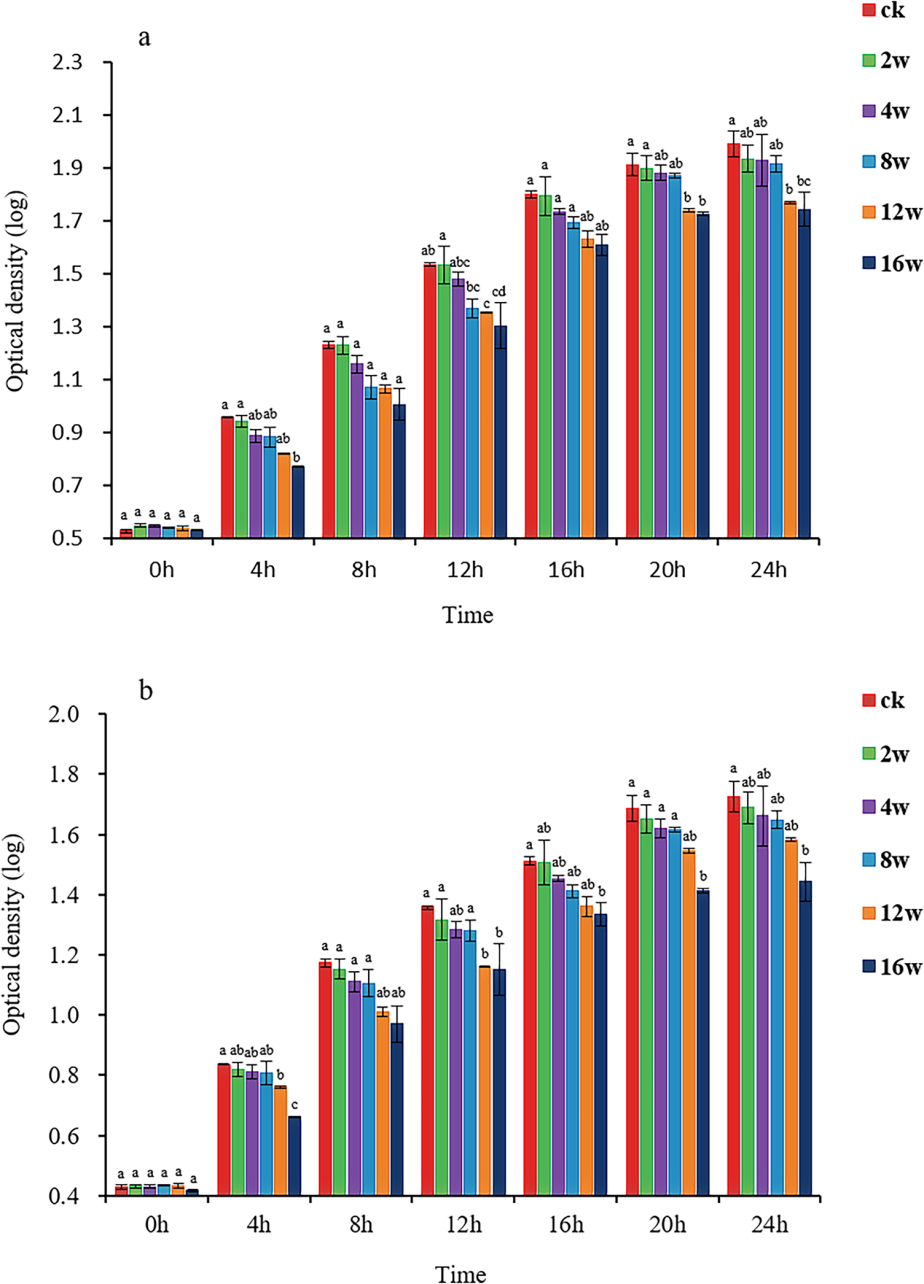
Antibacterial ability of oolong tea with different storage time. (**a**) Histogram of the antibacterial ability of oolong tea against Pathogenic *Escherichia coli.* (**b**) Histogram of the antibacterial ability of oolong tea against *Micrococcus tetragenus.* Columns with different color means oolong tea with different storage time. Ck means oolong tea without storage, 2w means oolong tea stored for 2 weeks, 4w means oolong tea stored for 4 weeks, 8w means oolong tea for 8 weeks, 12w means oolong tea stored for 12 weeks, and 16w means oolong stored for 16 weeks. The numbers on the abscissa represent incubation time of solution mixed with bacteria and tea infusion.

The antimicrobial activity of the tea sample was initially assessed by measuring the absorbance values of two bacterial solutions, where higher absorbance indicated greater bacterial activity. Conversely, lower absorbance denoted stronger antimicrobial effects. The changes observed in both bacterial solutions were similar. Initially, the tea samples exhibited a trend towards dynamic equilibrium during the 24-h period of the reaction (Fig. 1). Variances in absorbance values among the tea samples with different storage times were observed after the initiation of the reaction, with testing conducted every 2 h. Although an overall increase in absorbance values was noted, discrepancies were observed among the tea samples with varying storage times.

To provide enhanced visibility of the absorbance value changes among tea samples stored for different durations, a histogram was constructed (Fig. 1). Notably, the absorbance values of the tea samples were identical at 0 h. From 2 to 24 h, the absorbance values decreased with increasing storage time, indicating that the antibacterial potency of the tea strengthened with prolonged storage.

**Figure 2:**
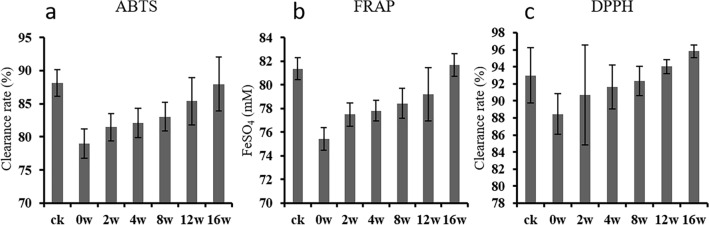
Antioxidant capacity of oolong tea with different storage time determined by different methods. (**a**) ABTS assay, (**b**) FRAP assay and (**c**) DPPH assay.

As the antimicrobial efficacy and antioxidant capacity of tea are commonly positively correlated, we also evaluated the antioxidative potential of different tea samples (Fig. 2). The outcomes from various tests for antioxidant capacity were consistent across the tea samples. It was observed that the antioxidant capacity of the tea samples increased with longer storage times, indicative of an improved antibacterial efficacy during the storage duration. Anjan Hazra et al.^49^ reported that antioxidant capacity of oolong tea exhibited constant decline during storage for 360 days at 25 °C. On the contrary, antioxidant capacity of oolong tea during storage for 16 weeks at 25 °C showed constant increase in this study perhaps because different type of oolong tea was used.

Numerous metabolites in tea have been previously demonstrated to possess both antibacterial and antioxidant properties^27,32,50^. Hence, our hypothesis postulates that the enhancement of tea's antibacterial efficacy during storage may be attributed to alterations in its constituents due to either oxidation or reduction processes. Moreover, it is evident from previous studies that degradation, microbial activity, and external factors can significantly impact the properties of tea^51–53^. Subsequently, we conducted the subsequent experiment to quantify the levels of amino acids, catechins, and alkaloids present in the tea sample.

### Quantitative changes in free amino acids during storage

To investigate the effort of storage on the content of free amino acids in WRT, a detailed analysis was conducted using a free amino acid analyzer. The results revealed the presence of a total of 23 individual free amino acids in WRT during storage. Upon initial analysis, the storage process demonstrated a highly significant effect (Hotelling multivariate p-value = 1 × 10^−6^). Subsequently, further statistical analyses were conducted to perform pairwise comparisons for the content of each free amino acid across all tea samples. The contents of the 23 individual free amino acids in the tea samples stored for different durations were presented in Table 1. It was worth noting that the total amino acid content steadily increases with storage duration, reaching its maximum at 16 weeks.

**Table 1:** Contents of 23 free amino acids in different tea samples (mg/g).

No.	Amino acid	ck	2w	4w	8w	12w	16w
1	Asp	5.04 ± 0.13e	5.35 ± 0.01d	5.88 ± 0.03c	5.44 ± 0.02d	6.12 ± 0.02b	6.36 ± 0.08a
2	Thr	1.08 ± 0.02e	1.23 ± 0.02d	1.36 ± 0.02c	1.28 ± 0.01d	1.42 ± 0.01bc	1.45 ± 0.02b
3	Ser	1.46 ± 0.01d	1.59 ± 0.01c	1.85 ± 0.01b	1.69 ± 0.02c	1.89 ± 0.01b	1.97 ± 0.04b
4	Glu	2.34 ± 0.02c	2.43 ± 0.02c	2.71 ± 0.01b	2.49 ± 0.02c	2.79 ± 0.01b	2.77 ± 0.02b
5	α-AAA	0.24 ± 0.01d	0.26 ± 0.01cd	0.31 ± 0.02bc	0.31 ± 0.01bc	0.36 ± 0.02a	0.35 ± 0.01ab
6	Gly	0.15 ± 0.01b	0.16 ± 0.01b	0.18 ± 0.01a	0.19 ± 0.00a	0.19 ± 0.01a	0.20 ± 0.01a
7	Ala	1.42 ± 0.01e	1.55 ± 0.03d	1.76 ± 0.01bc	1.69 ± 0.01c	1.82 ± 0.02ab	1.88 ± 0.03a
8	Cit	0.14 ± 0.01b	0.13 ± 0.01b	0.14 ± 0.01b	0.13 ± 0.01b	0.14 ± 0.01b	0.15 ± 0.01b
9	α-ABA	0.08 ± 0.00d	0.08 ± 0.01cd	0.09 ± 0.01bcd	0.09 ± 0.01bcd	0.10 ± 0.00bc	0.10 ± 0.01b
10	Val	1.15 ± 0.01d	1.32 ± 0.02c	1.46 ± 0.03b	1.36 ± 0.01c	1.54 ± 0.01a	1.55 ± 0.01a
11	Cys	0.93 ± 0.01c	0.99 ± 0.01c	1.14 ± 0.01b	1.01 ± 0.02c	1.14 ± 0.02b	1.12 ± 0.03b
12	Ile	0.91 ± 0.02d	0.98 ± 0.02c	1.15 ± 0.01ab	1.01 ± 0.01c	1.17 ± 0.01a	1.14 ± 0.02ab
13	Leu	0.72 ± 0.01e	0.84 ± 0.01d	0.93 ± 0.01bc	0.89 ± 0.03cd	0.95 ± 0.01bc	0.99 ± 0.03b
14	Tyr	1.99 ± 0.03d	2.21 ± 0.03c	2.54 ± 0.01b	2.32 ± 0.01c	2.71 ± 0.01a	2.66 ± 0.01ab
15	Phe	2.65 ± 0.04c	2.70 ± 0.01c	3.08 ± 0.02b	2.78 ± 0.02c	3.13 ± 0.03b	2.80 ± 0.04c
16	β-Ala	0.30 ± 0.01c	0.32 ± 0.01bc	0.37 ± 0.02bc	0.35 ± 0.02bc	0.40 ± 0.01b	0.39 ± 0.02b
17	GABA	1.08 ± 0.02e	1.25 ± 0.02d	1.35 ± 0.02c	1.27 ± 0.01d	1.43 ± 0.02b	1.47 ± 0.01b
18	Om	0.45 ± 0.01d	0.61 ± 0.02c	0.66 ± 0.02c	0.76 ± 0.03b	0.78 ± 0.01b	0.94 ± 0.03a
19	His	0.22 ± 0.01b	0.24 ± 0.02b	0.32 ± 0.02a	0.27 ± 0.01b	0.32 ± 0.02a	0.31 ± 0.01a
20	Arg	0.55 ± 0.02f	0.70 ± 0.02de	0.83 ± 0.01b	0.73 ± 0.01cd	0.81 ± 0.03bc	0.96 ± 0.02a
21	Pro	0.67 ± 0.14b	0.87 ± 0.03b	0.94 ± 0.01a	0.97 ± 0.04a	1.04 ± 0.03a	1.09 ± 0.04a
22	Gln	0.98 ± 0.01b	1.14 ± 0.05b	1.21 ± 0.05b	1.22 ± 0.06b	1.25 ± 0.06b	1.43 ± 0.05b
23	Thea	16.85 ± 0.34d	16.65 ± 0.97c	16.35 ± 1.06c	16.05 ± 1.13c	16.01 ± 1.13c	15.92 ± 0.74b
	Total	41.39	43.63	44.62	46.31	47.53	47.97

*Asp* aspartic acid, *Thr* l-threonine, *Ser* l-serine, *Glu* l-glutamic acid, *α-AAA* alpha-aminoadipic acid, *Gly* glycine, *Ala* l-alanine, *Cit* citrulline, *α-ABA* α-amino-*n*-butyric acid, *Val* l-valine, *Cys* l-cysteine, *Ile* l-isoleucine, *Leu* l-leucine, *Tyr* l-tyrosine, *Phe* l-phenylalanine, *β-Ala* β-alanine, *GABA* γ-aminobutyric acid, *Orn* l-ornithine, *His* l-histidine, *Arg* l-arginine, *Pro* l-proline, *Gln* glutamine, *Thea* theanine, *ND* not detected.

Means (± SD) labeled with different letters within each row are significantly different by ANOVA (p = 0.05, n = 3).

**Figure 3:**
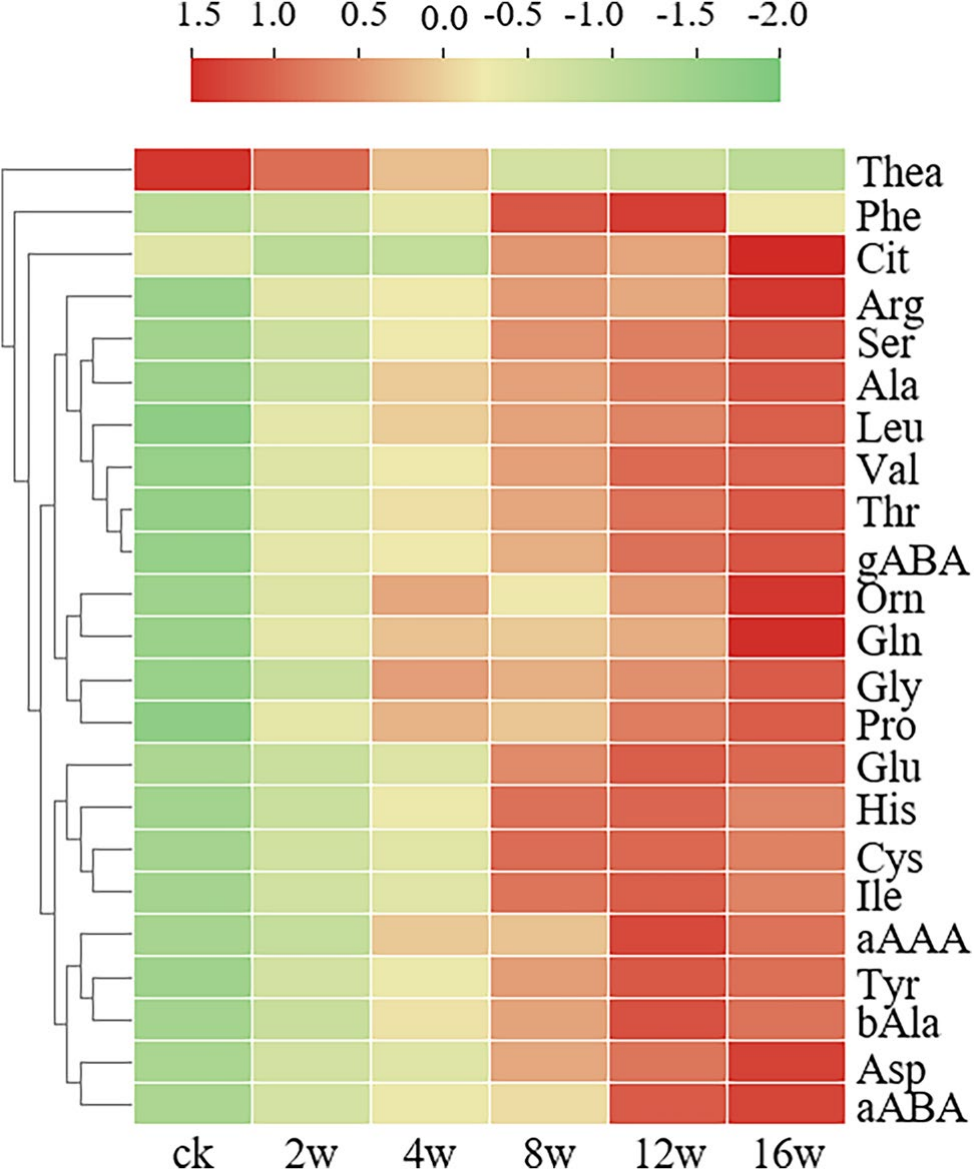
Heatmap of free amino acids in oolong tea with different storage time. *Thea* theanine, *Phe* l-phenylalanine, *Cit* citrulline, *Arg* l-arginine, *Ser* l-serine, *Ala* l-alanine, *Leu* l-leucine, *Val* l-valine, *Thr* l-threonine, *gABA* γ-aminobutyric acid, *Orn* l-ornithine, *Gln* glutamine, *Gly* glycine, *Pro* l-proline, *Glu* l-glutamic acid, *His* l-histidine, *Cys* l-cysteine, *Ile* l-isoleucine, *α-AAA* alpha-aminoadipic acid, *Tyr* l-tyrosine, *bAla* β-alanine, *Asp* aspartic acid, *α-ABA* α-amino-n-butyric acid.

For a more comprehensive analysis of the changes in free amino acids detected during tea storage, a heat map illustrating the amino acid quantities in the various tea samples was generated (Fig. 3). In the figure, red indicates higher content, while green indicates lower content. Comparing the variation in color intensity among the samples revealed that different storage times of oolong tea had distinct effects on individual free amino acids.

For individual free amino acid, different variation trends were observed in Fig. 3. Concentration of Thea decreased over time. The levels of Phe exhibited an initial increase until 12 weeks, followed by a decrease at 16 weeks. Furthermore, the content of Cit experienced a slight decline at 2 weeks and 4 weeks. In contrast, the remaining 20 free amino acids consistently increased with storage duration and demonstrated significant variation across different time points. These findings suggest that diverse physical and physiological reactions, as well as subsequent Strecker degradation, occurred in various tea samples during storage, leading to alterations in the free amino acid content in different tea types.

The amino acids Thea, Asp, Gln, GABA, Arg, Tyr, Phe, Ala, Glu, Ser, and Thr exhibited significantly higher levels compared to other amino acids in all tea samples (Table 1 and Fig. 3). Thea emerged as the predominant amino acid in tea, with consistent content levels ranging from 15.92 to 16.85 mg/g, thus indicating its prominence among all amino acids. This phenomenon is likely influenced by microorganisms and the tea conversion process. Notably, Thea, Glu, Asp, Cys, and Met have been identified as the primary contributors to the umami taste of tea infusions^54^. Jiakun Peng et al.^55^ reported that most amino acids in oolong tea show decrease trend after stored for up to 15 years. However, the content of amino acids except Thea increased during the storage of 16 weeks in this study because of relative short storage period.

**Figure 4:**
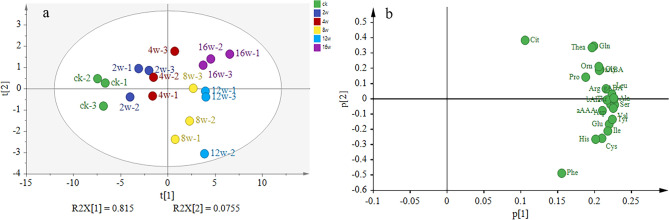
PCA score plot and loading plot of free amino acids in tea samples during storage. (**a**) PCA score plot of free amino acids in tea samples during storage. (**b**) Loading plot of free amino acids in tea samples during storage.

Using principal component analysis (PCA), this study further investigated variations in the free amino acid content at different storage durations (Fig. 4). Principal components 1 and 2 accounted for 81.5% and 7.5% of the total variance, respectively. Notably, distinct differences were observed among the seven groups in the PCA score plot (Fig. 4a). These findings suggest that the free amino acid content is effective in distinguishing oolong tea during storage. In the PCA loading plot (Fig. 4b), a wide array of free amino acids is depicted, indicating that Thea, Gln, Phe, and Cit were the primary contributors to differentiating tea samples.

The free amino acid profiles, in combination with a multivariate statistical method, could effectively classify tea samples based on their storage durations, aligning with prior research indicating the discriminative potential of amino acids across tea types^56^. Further research utilizing multivariate statistics is warranted, encompassing an exploration of other tea constituents for discriminating various types of tea during storage.

### Quantitative changes in catechins and alkaloids during storage

Table 2 displayed the quantitative data for the catechins, alkaloids, and phenolic acids in the tea samples. The total catechin content exhibited an increase with prolonged storage. The content of EGCG and CAF reached peak levels and exhibited an increase over the storage duration, suggesting a beneficial impact of tea storage on their levels. The bitter-tasting CAF, a prominent alkaloid in tea, is linked to diverse health benefits^57^. EGCG is the major catechin and makes up over 10% of the dry weight of green tea^58^. Its powerful antioxidant activity and health care effects have received much attention in recent years.

**Table 2:** Contents of catechins and alkaloids in different tea samples.

No.	Compounds	Contents (mg/g)					
		ck	2w	4w	8w	12w	16w
1	GA	0.19 ± 0.01a	0.18 ± 0.01a	0.18 ± 0.01a	0.17 ± 0.01a	0.18 ± 0.01a	0.18 ± 0.01a
2	GC	8.89 ± 0.05b	9.21 ± 0.13a	9.19 ± 0.05a	9.03 ± 0.11ab	8.98 ± 0.13ab	9.03 ± 0.05ab
3	THB	0.30 ± 0.01a	0.29 ± 0.02a	0.29 ± 0.01a	0.29 ± 0.01a	0.31 ± 0.01a	0.31 ± 0.01a
4	EGC	23.98 ± 0.22e	25.62 ± 0.23c	25.81 ± 0.03c	22.77 ± 0.08f	24.77 ± 0.02d	26.41 ± 0.25b
5	C	0.62 ± 0.01d	0.64 ± 0.01d	0.78 ± 0.01c	0.82 ± 0.01b	0.84 ± 0.01b	0.88 ± 0.02a
6	EGCG	34.08 ± 0.13f	35.56 ± 0.32ef	36.4 ± 0.42de	37.65 ± 1.07cd	39.30 ± 0.42bc	39.77 ± 0.58b
7	CAF	34.28 ± 0.18d	34.93 ± 0.39cd	35.57 ± 0.14bcd	36.11 ± 0.73bc	36.82 ± 0.96ab	38.26 ± 0.22a
8	EC	6.30 ± 0.17bc	6.73 ± 0.52ab	5.90 ± 0.05c	5.96 ± 0.10c	6.20 ± 0.09bc	5.99 ± 0.14bc
9	ECG	14.52 ± 0.26b	14.01 ± 0.94b	12.42 ± 0.02c	13.28 ± 0.15bc	13.12 ± 0.1bc	13.63 ± 0.39bc
	Total catechins	123.16	127.17	126.54	126.08	130.52	134.46

*GA* gallic acid, *THB* theobromine, *EGC* (−)-epigallocatechin, *C* (+)-catechin, *CAF* caffeine, *EC* (−)-epicatechin, *EGCG* (−)-epigallocatechin gallate, *GC* (−)-gallocatechin gallate, *ECG* (−)-epicatechin gallate.

Means (± SD) labeled with different letters within each row are significantly different by ANOVA (p = 0.05; n = 3).

**Figure 5:**
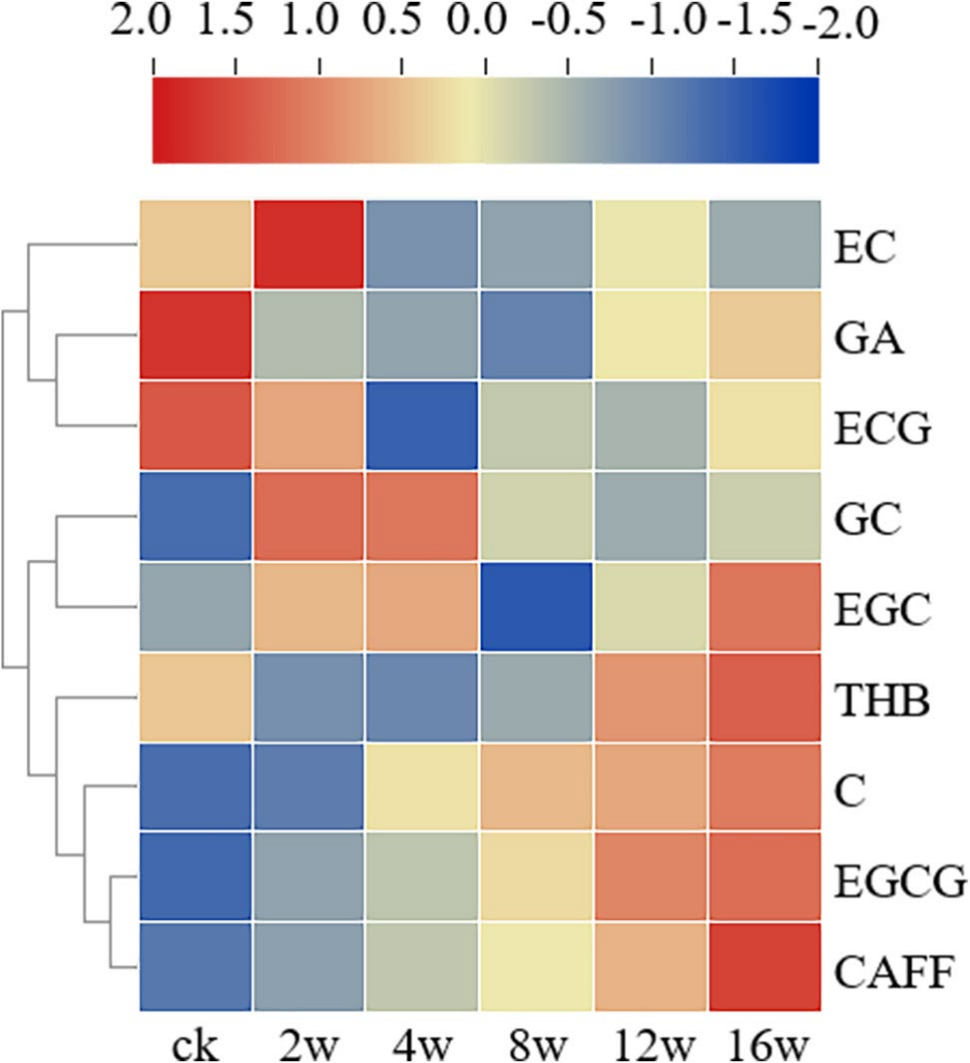
Heatmap of the content of catechins and alkaloids in tea samples during storage.

The patterns of change differed among compounds during the storage of tea. A heat map presents the content of each catechin and alkaloid in the tea samples during storage (Fig. 5). With the exception of the increased content of EGCG and CAF over time, there was a slight increase in the content of C, while all other catechins and alkaloids exhibited no apparent change.

The findings of this investigation suggest that the regular alterations in certain primary components among catechins and alkaloids could be influenced by the duration of tea storage, potentially affected by the storage conditions and microbial activity. In summary, the variations in catechins and alkaloids can serve as indicators of changes in the quality of tea leaves over the storage period.

### Relationship between the antibacterial effect of tea and metabolites

**Figure 6:**
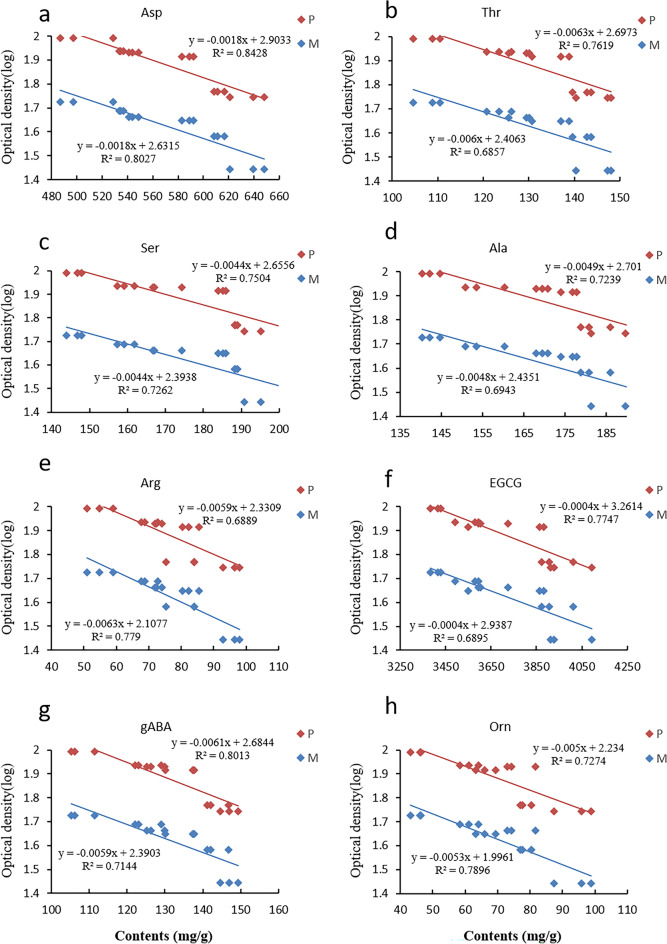
Correlation between certain metabolites and their antibacterial activity against Pathogenic *Escherichia coli* (P, red) and *Micrococcus tetragenus* (M, blue). (**a**) Asp, aspartic acid; (**b**) Thr, l-threonine; (**c**) Ser, l-serine; (**d**) Ala, l-alanine; (**e**) Arg, l-arginine; (**f**) EGCG, Epigallocatechin gallate; (**g**) gABA, γ-aminobutyric acid; (**h**) Orn, l-ornithine. The abscissa represents the content of metabolites, and the ordinate means the log-transformed optical density of solution mixed with different tea infusion and bacterial culture solution. Higher optical density indicates higher bacterial activity and lower antibacterial activity of metabolite.

To investigate the factors contributing to the variations in the antibacterial efficacy of tea during storage, we conducted a correlation analysis between the antibacterial activity and contents of bioactive compounds in tea infusion. Different reaction times were employed to test the tea samples at various storage durations. Notably, the absorbance values varied, revealing a correlation between antibacterial activity and metabolites. Furthermore, prominent markers of antibacterial efficacy displayed similar trends in the data at each time point, as illustrated in Fig. 1, thereby justifying the correlation analysis of the antibacterial activity and tea sample metabolites specifically at the 24-h mark. Remarkably, the bacteriostatic ability against *M. tetragenus* showed significant correlations (R^2^ > 0.7) with several metabolites, including Asp, Ser, GABA, aABA, Val, Tyr, Orn, catechins, alkaloids, and CAF throughout the 24-h reaction. Moreover, the antibacterial efficacy against pathogenic *E. coli* in the 24-h reaction demonstrated significant correlations with Glu, Asp, Ser, Ala, GABA, Orn, Arg, catechins, alkaloids, and EGCG. Thus, it is evident that amino acids significantly influenced by the metabolites (R^2^ > 0.68) regardless of bacterial Gram classification were Asp, Thr, Ser, GABA, Orn, Ala, Arg, and EGCG, as depicted in Fig. 6. EGCG have demonstrated broad antimicrobial spectrum such as antifungal, antibacterial and antiviral effects^59^, and the increase of EGCG content was the main reason for the improvement of antibacterial activity of oolong tea after storage.

## Conclusion

The tea samples underwent quantitative analysis of catechin, free amino acid, and alkaloid content at various storage times through a rapid analytical method. Subsequent scrutiny of the fluctuations in these components aimed to gauge the impact of diverse storage durations on the primary chemical constituents of tea. PCA and heat map analysis were employed to identify pivotal substances, such as EGCG, theanine, and glutamine, which exhibited notable susceptibility to the storage process. These identified markers hold potential for the tea industry to facilitate quality surveillance of oolong tea during storage. Notably, the antibacterial potency of tea escalated with prolonged storage, evidenced by correlation analysis pinpointing the influence of metabolites Asp, Thr, Ser, GABA, Orn, Ala, Arg, and EGCG on the antibacterial efficacy against the two bacteria assessed. In summary, this investigation underscores the utility of certain metabolites in overseeing both the quality and antibacterial effectiveness of tea during storage.

## Data Availability

All data used for this study are contained in this article.
